# Metabolic shift in sugars and amino acids regulates sprouting in Saffron corm

**DOI:** 10.1038/s41598-017-10528-2

**Published:** 2017-09-19

**Authors:** Jayram Bagri, Anupama Yadav, Khalid Anwar, Jeremy Dkhar, Sneh Lata Singla-Pareek, Ashwani Pareek

**Affiliations:** 1 Stress Physiology and Molecular Biology Laboratory, School of Life Sciences, Jawaharlal Nehru University, New Delhi, India; 2 Plant Stress Biology, International Centre for Genetic Engineering and Biotechnology, Aruna Asaf Ali Marg, New Delhi, India

## Abstract

Saffron is one of the most expensive spices of the world. Since this spice is triploid and meiosis is unusual, it cannot reproduce sexually like other plants; rather, it is propagated vegetatively via an underground corm, which can withstand a long dry dormant period before sprouting. Thus, corms are indispensable to saffron propagation. To identify and analyse signature metabolites associated with the ‘dormancy-sprouting’ process, non-targeted GC-MS was performed at different stages of corm development. Comparative metabolite profiling reflected dissimilar profiles among the stages as portrayed by differential cluster patterns of metabolites in the PCA and PLS-DA analysis. Correlation analysis revealed the interdependencies of individual metabolites and metabolic pathway. At the onset of stage 2, characterized by the initiation and differentiation of leaf primordia, a shift from dormancy to active metabolism occurred as derived from the increased abundance of sugars and other metabolites involved in the tricarboxylic acid cycle, glycolytic, amino acid and fatty acid pathways. These changes contribute to sprouting and vegetative growth of the corm. The present study provides new insights into saffron corm composition and metabolite changes associated with various stages of corm development and may pave the way for achieving agronomical improvements in this economically important spice.

## Introduction

Saffron (*Crocus sativus* L.) is one of the most expensive and elusive spices of the world, grown in areas where Mediterranean type of climate prevails. In India, large-scale cultivation is restricted to the state of Jammu and Kashmir, and this contributes significantly to the economy of the state^1, 2^. It is mostly used as a food condiment with limited usage in textile or perfumery. Due to its analgesic and sedative properties, folk herbal practitioners have also used saffron for the treatment of several ailments^3, 4^.

Saffron is a triploid perennial sterile plant that can withstand dry dormant period, a characteristic attributed to its underground corm^5–7^. Thus, corms are indispensable to saffron propagation, surviving for only a season and reproducing vegetatively into cormlets that ultimately develop into new plants. Saffron plants remain as corms at the start of the dry season (April-May, when the leaves begin to senesce and wither) until the end of summer (July), when the initiation of leaf primordia is observed^8, 9^. Flower morphogenesis soon takes place and fully developed flower can be seen in the fields by the end of October. With the onset of sprouting, the corms become a source organ sustaining the growth of the newly developing corms^8^. For the newly developed ones to produce flowers, corms should attain an appropriate size^10^. Therefore, production of corms of adequate size is extremely important to ensure flower production. At the same time, determining the factors affecting the sprouting process is highly essential for corm and flower production^11^. Apical buds formed on a mature saffron corm sprout in the subsequent season along with many other axillary dormant buds^12^. Following the development of a few leaves, the axillary buds enter into a dormant stage. These axillary buds are believed to offer rapid recovery from damage to the plant and to adjust its growth in accordance with the environmental stimuli^10, 12^. Current conventional practices allow production of only two to three corms per mother corm^13^. Thus, an initial corm would require about 9-10 years to produce corms sufficient for sowing in one hectare of land^14^. Besides the low multiplication rates, corm productivity and quality is further reduced by fungal diseases, thereby restraining the availability of the planting material. Erratic and scanty rainfall, which is distributed irregularly, further impose adverse effect on the production of saffron flower^15^. The state of Kashmir in India experienced an acute drought during 1999-2003, which resulted in reduced productivity of Saffron from 3.0 kg/ha to 1.5 kg/ha^16^.

Attempts have been made to understand the ‘dormancy-sprouting’ process in saffron corm. Using the boiling ethanol (80%) method to separate starch and total sugars from chopped saffron corm tissues, Chrungoo and Farooq^17^ found no significant change in both starch and total sugar contents during dormancy. However, as sprouting progresses, starch accumulation decreases progressively whereas total soluble sugars content increases gradually^17^. These findings suggest a shift in metabolite accumulation as the saffron corm undergoes ‘dormant-sprouting’ process. This pattern of metabolic shift in starch and sugar contents was also observed during the ‘dormant-sprouting’ process in the tubers and rhizomes of *Dioscorea esculenta* (yam) and *Curcuma longa* (turmeric), respectively^18^. More recently, Jain *et al*.^19^, performed an RNA-seq based transcriptomic study and showed that transcripts encoding key enzymes involved in starch degradation, plant hormone biosynthesis and flavonoid biosynthesis are highly enriched in the dormant corm. In fact, Rubio-Moraga *et al*.^11^ showed that the plant hormone auxin is present at high levels in the apical bud, probably to inhibit growth of axillary buds. This increased accumulation of auxin is correlated with an increased expression of a carotenoid cleavage dioxygenase CCD8, a gene involved in the production of strigolactones. This study shows that auxin and strigolactones act synergistically to suppress growth of the axillary bud from saffron corm.

The ‘dormancy-sprouting’ process in plants is orchestrated by a complex interaction of phytohormones abscisic acid (ABA) and gibberellin (GA). These phytohormones have also been documented to be correlated with the onset and upholding of corm dormancy (endo-dormancy) and apical dominance (para-dormancy)^20, 21^. Besides phytohormones, primary metabolites also play an important role, at least in the sprouting process. For example, sprouting in mung bean (*Vigna radiata*) is associated with increased levels of monosaccharides, organic acids and amino acids and a decrease in fatty acid methyl esters level^22^. Till date, little is known about the direct role or association of primary metabolites (sugars, amino acids, organic acids, and fatty acids) in the ‘dormancy-sprouting’ process of *Crocus* corm. A complete dissection of the ‘dormancy-sprouting’ process is thus needed to gain insight into the events underlying this crucial process.

Recent advances in analytical techniques have facilitated an in-depth analysis of metabolites present in a given tissue. Metabolites are usually considered as the biochemical phenotype of an organism, as they represent the end products of gene expression^23, 24^. About 200,000 different metabolites are present in plants^25^; plant metabolomics seek to identify and quantify these metabolites using various analytical techniques such as gas chromatography – mass spectrometry (GC-MS)^26, 27^. GC-MS has been used for the identification and quantification of metabolites in several plant species such as *Glycine max* ^28^, *Vigna radiata* ^22^, *Solanum lycopersicum* cv.^29^ and *Hordeum vulgare* ^30^.

**Figure 1:**
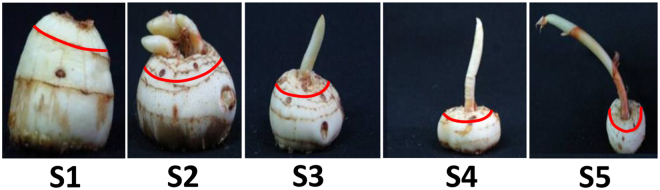
Dormancy and sprouting in Saffron corm (*C. sativus* L.). Saffron corm at key developmental stages are shown: S1 represents the dormancy period (early May-July), S2 depicts the initiation and differentiation of leaf primordia (early July-August), S3 shows the initiation and differentiation of floral primordia (early August -late August), S4 denotes elongation of perianth tube (late August-early October), while S5 indicates bud break and floral anthesis (early October- late October). Red circle in each picture represents the apical bud portion from where tissue was taken for the present study.

To dissect out the complex spatial changes in primary metabolites involved in dormancy (endo-dormancy) and sprouting process of Saffron corm, we carried out, for the first time, GC-MS based profiling of primary metabolites at five distinctly identified stages of corm development viz. stage 1 (S1) – quiescent; stage 2 (S2) – initiation and differentiation of leaf primordia; stage 3 (S3) – initiation and differentiation of floral primordia; stage 4 (S4) – elongation growth of perianth tube and stage 5 (S5) – bud break and floral anthesis (Fig. 1).In the present work, we report a progressive increase in the level of total sugars, specifically glucose and fructose with a simultaneous decline in sucrose during transition from S2 to S3. This observation clearly suggest that reducing monosaccharides finally reach their highest relative abundance, as a result of sucrose breakdown and initiate the sprouting process and bud growth in Saffron corm.

## Results

### GC-MS based metabolite profiling of saffron corm at key developmental stages

**Figure 2:**
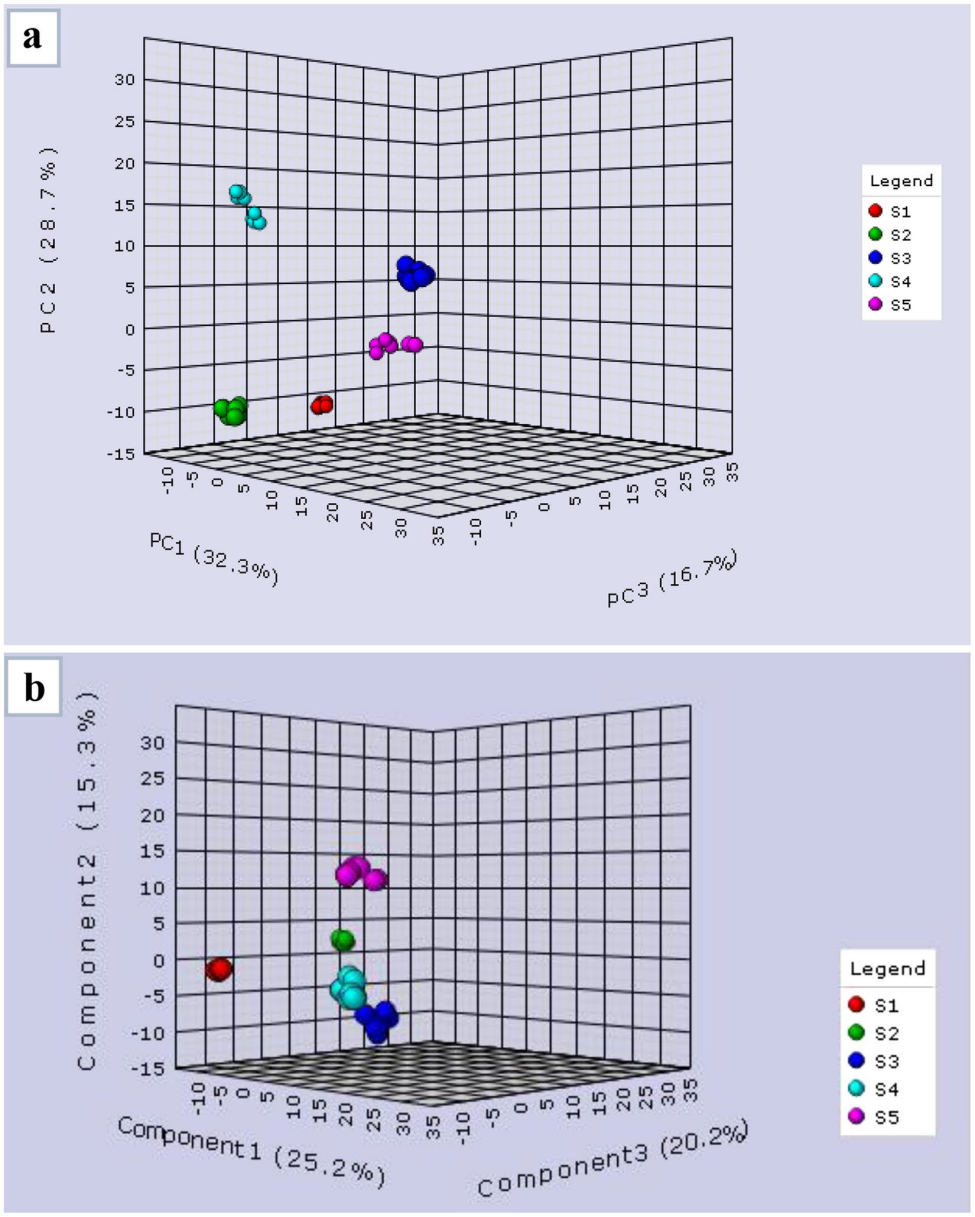
(**a**) Principle component analysis (PCA) of metabolite profiles of distinct developmental stages of Corm (S1-S5) during dormancy and sprouting process. PCA presented as combination of first three dimensions, which together comprise 78.7% of the metabolite variance. (**b**) Partial least square-discrimination analysis (PLS-DA) of metabolite profiles of distinct developmental stages (S1–S5) during dormancy and sprouting process. PLS-DA presented as combination of first three dimensions, which together comprise 60.7% of the metabolite variance. A web-based tool MetaboAnalyst 2.0 was used to analyse the data. The separate percentages of the variance for each plot are given in brackets in each section.

To unravel the differential metabolome of the developing corm of Saffron, five distinct stages of corm were analysed by GC-MS using eight biological replicates for each stage. In total, 89 known metabolites that include amino acids, sugars, sugar alcohols, organic acids, and fatty acids were obtained from the GC-MS analysis of tissue obtained from five different stages of corm development (Table S1, Supplementary information). Among them, a number of metabolites were also identified that showed exclusive accumulation in certain stages of corm development. Of relevance to the sprouting process, leucine, ornithine, oleic acid and lactic acid were exclusively detected in stages 2 and 3. The identified metabolites are involved in various metabolic pathways such as tricarboxylic acid cycle (TCA), glycolysis, amino acid biosynthesis, fatty acid biosynthesis, organic acid biosynthesis and sugar biosynthesis. The highest number of known metabolites was detected in the tissue belonging to stage3 (62), followed by S2 (58), S5 (39), S4 (38) and then S1 (37), (Table S1, Supplementary information). Around 20% metabolites were common among all the five developmental stages analysed here. Multivariate statistical analysis of metabolite data through PCA showed clear discrimination among the five stages of corm analysed here (Fig. 2a). Similarly, PLS-DA of metabolites at various developmental stages showed separate clustering for each stage (Fig. 2b). The data is very robust as evident from the close clustering observed in the eight biological replicates of each stage presented here.

**Figure 3:**
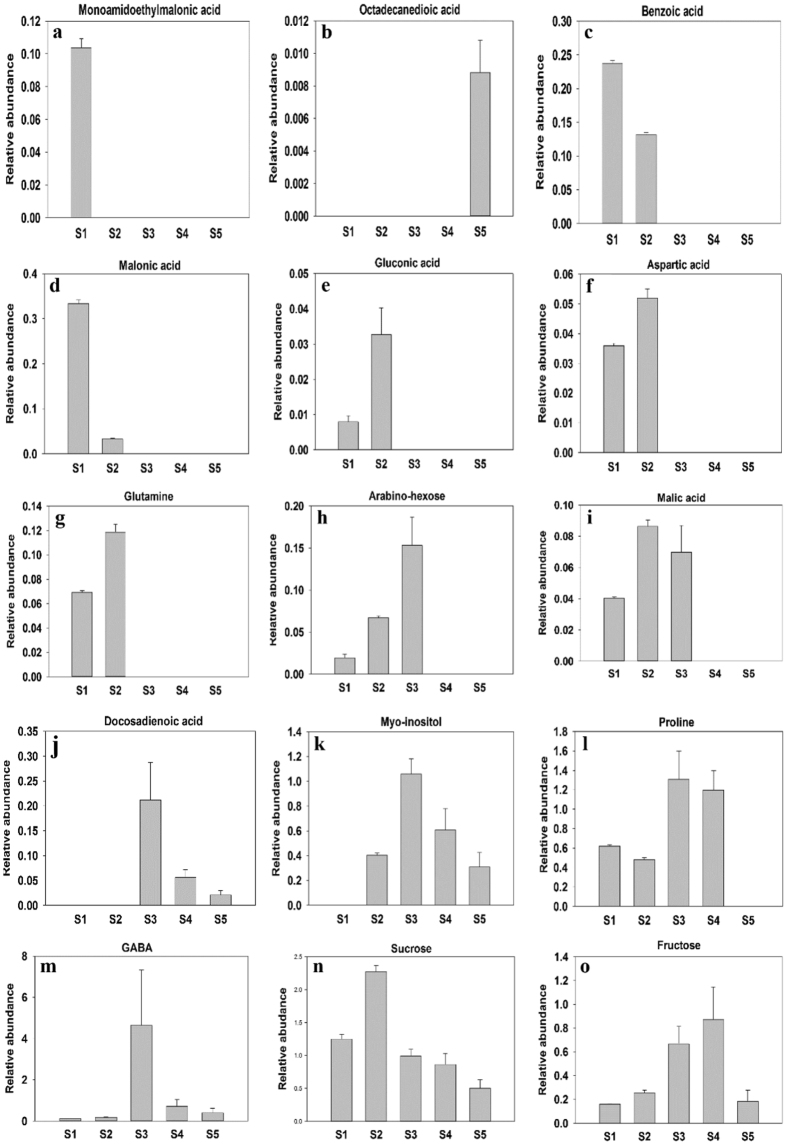
Graphical representation of metabolites identified from the PLS-DA variable importance projection (VIP) showing major differences in accumulation in Saffron corm at five different developmental stages (S1–S5).

A total of 15 signature metabolites playing a significant role in PLS-DA clustering were further identified and their relative abundance pattern at each stage was examined (Fig. 3 and Fig. S1, Supplementary information). We found two metabolites viz. monoamidoethylmalonic and octadecanedoic acid showing exclusive accumulation at stages 1 and 5, respectively (Fig. 3a,b). Benzoic acid and malanoic acid showed increased accumulation at stage S1 as compared to S2 but could not be detected in tissues of other stages (Fig. 3c,d). On the contrary, gluconic acid, aspartic acid and glutamine showed higher abundance at S2 as compared to S1, with no accumulation in S3-S5 tissues (Fig. 3e,f,g). Malic acid and arabino-hexose were detected in the tissue from first three stages. Unlike malic acid which showed successive increase in accumulation from S1 to S3, arabino-hexose accumulation increased from S1 to S2 but decreased at S3 (Fig. 3h,i). Docosadienoic acid started accumulating at stage 3, but got reduced at stages 4 and 5 (Fig. 3j). Myo-inositol and proline were detected at four stages of corm development. The accumulation of myo-inositol begins at S2, increases further at S3 but lowers down in S4 and S5 (Fig. 3k). Proline, on the other hand, was detected in the tissue from first four stages with increased accumulation in S3 and S4 (Fig. 3l). Three other metabolites viz. GABA, sucrose and fructose were detected at all five stages with varying pattern of accumulation. GABA is most abundant at S3 with relatively lower accumulation at the other stages whereas sucrose showed maximum abundance at S2 (Fig. 3m,n). The accumulation of fructose progresses from S2-S4, but abruptly reduces in S5 (Fig. 3o).

**Figure 4:**
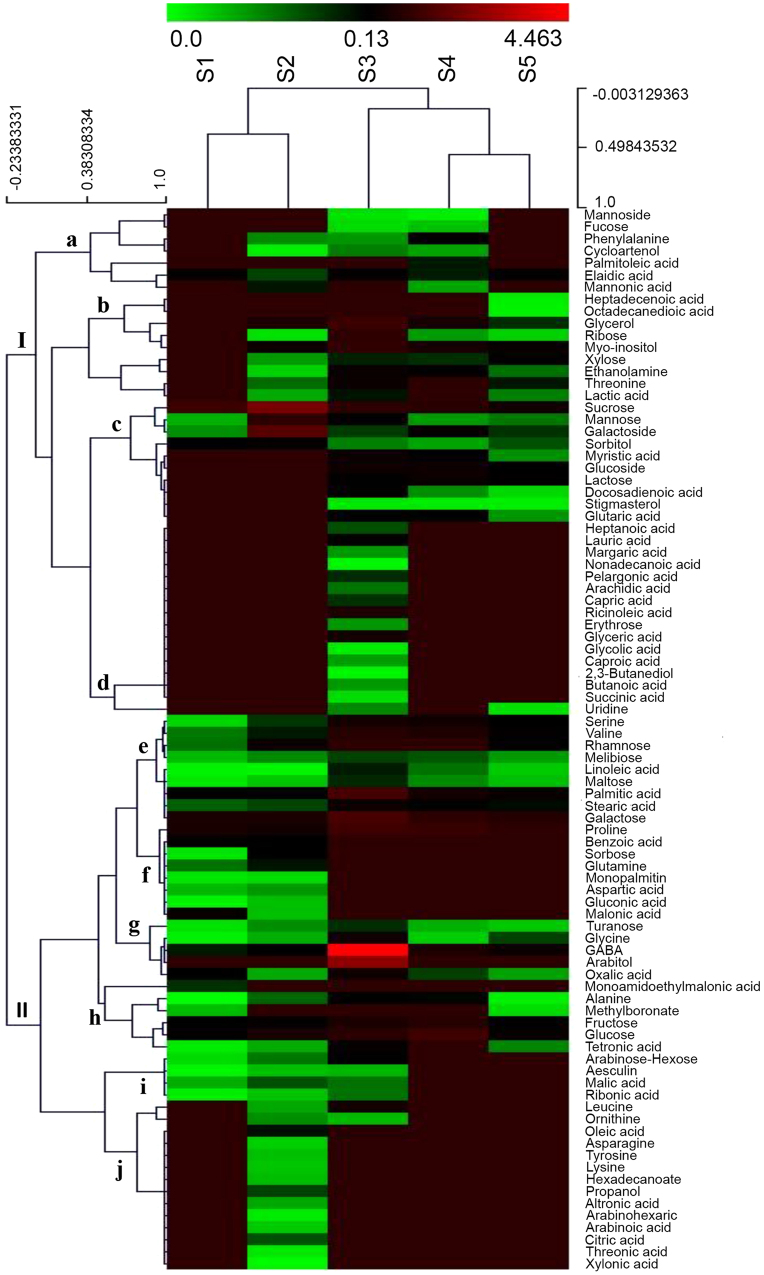
A hierarchically clustered heat map of the primary metabolite levels of 89 representative metabolites at different developmental stages of saffron corm. The relative values of each metabolite were normalized to the internal standard ribitol of each developmental stage and are provided in Table S1, Supplementary information. Dark green represents lowest accumulation while the highest accumulation is depicted as dark red. S1–S5 refers to the five developmental stages of *Crocus* corm.

Hierarchical clustering of the 89 metabolites depicted two major clusters (Fig. 4). Cluster I is further divided into four sub-clusters (a–d) while Cluster II showed 6 sub-clusters (e–j). Cluster I comprises metabolites showing increased accumulation at the initial stages. Metabolites of sub-cluster ‘d’ showed accumulation at the later stages as well. On the contrary, majority of metabolites of Cluster II showed accumulation at the later stages of corm development (Fig. 4).

### Metabolite changes during transition from dormant to reproductive stage of Saffron corm

**Figure 5:**
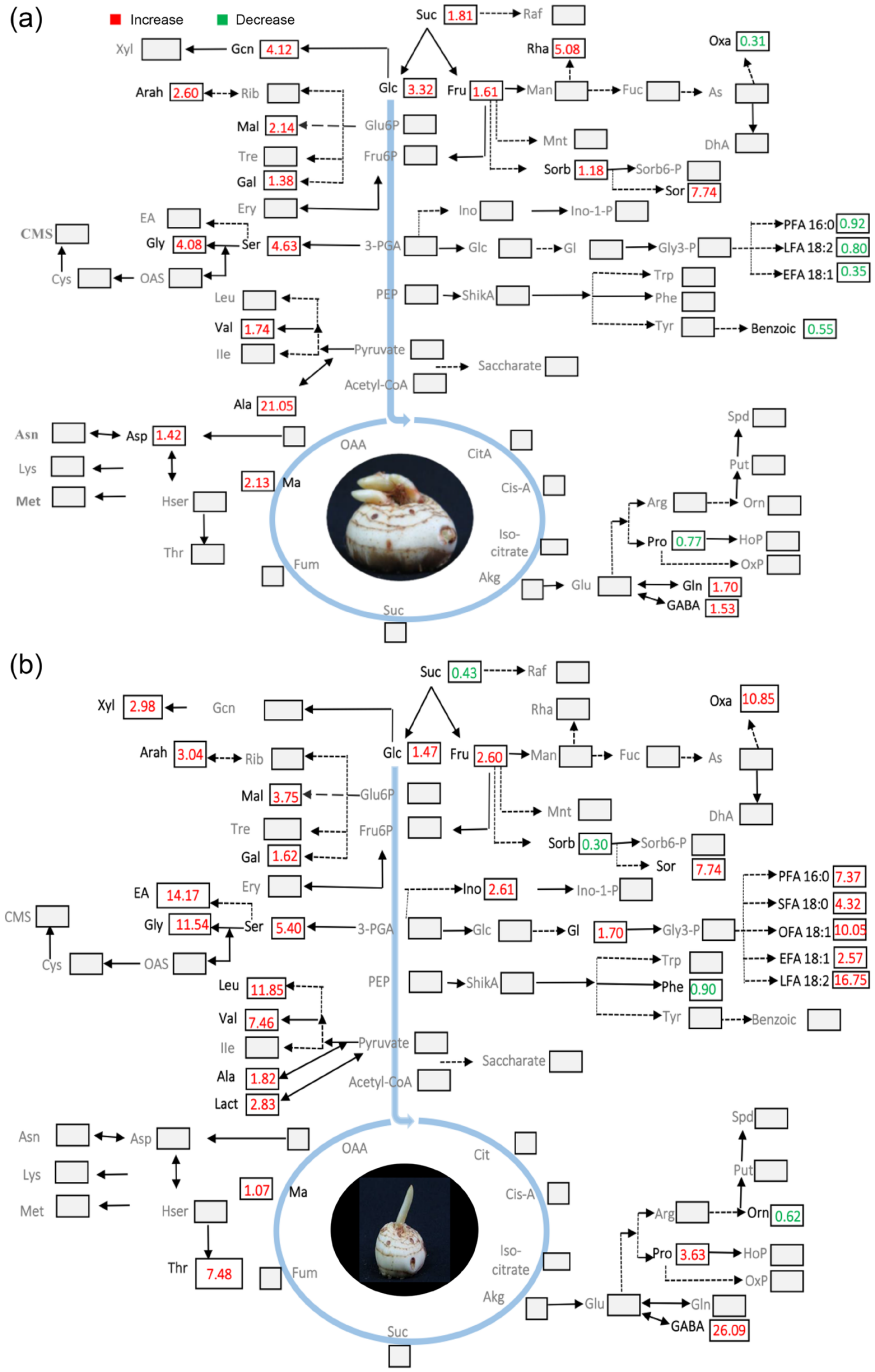
Metabolic map depicting fold change (P < 0.05) in metabolites between S2 and S1 (**a**) and S3 and S2 (**b**) developmental stages of *Crocus* corm. Undetected metabolites are denoted by grey characters. The metabolites identified in this study are: Alanine (Ala); Aspartate (Asp); Arabino-hexose (Arah); Elaidic acid (EFA18:1); Ethanolamine (EA); Fructose (Fru); Galactose (Gal); Gamma-aminobutyric acid (GABA); Glucose (Glc); Glutamine (Gln); Glycine (Gly); Glyceric (Glc); Gluconic acid (Gcn); Lactic acid (Lact); Leucine (Leu); Linoleic acid (LFA18:2); Myo-inositol (Ino); Maltose (Mal); Malic acid (Ma); Oleic acid (OFA18:1); Ornithine (Orn); Oxalic acid (Oxa); Palmitic acid (PFA16:0); Phenylalanine (Phe); Proline (Pro); Ribose (Rib); Serine (Ser); D-Sorbitol (Sorb); Sorbose (Sor); Stearic acid (SFA18:0) Sucrose (Suc); Threonine (Thr); Valine (Val); Xylose (Xyl).

To identify the metabolic pathways that modulate the transition from dormant to sprouting stage, a pair-wise comparison between all the stages was performed. We observed significant metabolite changes (P < 0.05) between S1 and S2 as depicted on the metabolic map (Fig. 5a). In S2 (initiation and differentiation of leaf primordia), we observed significant increase in sucrose (1.81 fold), sorbose (7.74 fold), rhamnose (5.08 fold), glucose (3.32 fold), galactose (1.38 fold), maltose (2.14 fold), sorbitol (1.18 fold), fructose (1.61 fold), arabino-hexose (2.60 fold), turanose (3.45 fold), glycine (4.08 fold), serine (4.63 fold), valine (1.74 fold), aspartic acid (1.45 fold), glutamine (1.70 fold), GABA (1.53), alanine (21.05 fold), malic acid (2.13 fold), gluconic acid (4.12) and ribonic acid (2.77 fold) accompanied by significant decrease in stearic acid (0.88 fold), oxalic acid (0.31 fold), proline (0.77 fold), and benzoic acid (0.55 fold), elaidic acid (0.35 fold), linoleic acid (0.80 fold) and palmitic acid (0.92 fold) (Fig. 5a).

We also observed significant metabolite changes (P < 0.05) between stages 2 and 3. In S3 (initiation and differentiation of floral primordia), we observed significant increase in glycine (11.54 fold), serine (5.40 fold), valine (7.46 fold), GABA (26.09 fold), leucine (11.85 fold), proline (3.36 fold), threonine (7.48 fold), ethanolamine (14.17 fold), alanine (1.82 fold) glycerol (1.70 fold), lactic acid (2.83 fold), myo-inositol (2.61 fold), oleic acid (10.05 fold), stearic acid (4.32 fold), linoleic acid (16.75 fold), palmitic acid (7.37 fold), oxalic acid (10.85 fold), glucose (1.47 fold), malic acid (1.07 fold), arabino-hexose (3.04 fold), elaidic acid (2.57) fructose (2.60 fold), maltose (3.75 fold), xylose (2.98 fold) and galactose (1.62 fold) with significant decrease in phenylalanine (0.90 fold), ornithine (0.62) sucrose (0.43 fold) and sorbitol (0.30 fold) (Fig. 5b).

During development through S4, metabolite levels gradually dropped and reached minimum level at S5, resulting in an almost complete reversal of the metabolic changes. The levels of major amino acid, fatty acids, sugars and organic acids showed a significant decrease. A significant decrease in the level of sucrose was also observed during transition from S4 while the level of fructose and glucose were detected at S4 and S5, which suggested that the catabolism of sucrose occurred during sprouting process of Saffron corm and it might play a significant role during progression from dormant to reproductive stage of Saffron corm.

### Correlation analysis of metabolites during development in Saffron corm

Correlation analysis has become a useful tool in investigating metabolic pathways and networks^26, 27^. To define metabolic changes related to sprouting process of Saffron corm, the pairwise Pearson correlation coefficient was analysed for each metabolite at different development stages against every other metabolites within the sample. We treated metabolites with correlation coefficients ^r^Met ≥ 0.8 as strong dependencies. We categorized correlation pairs of metabolites according to their placement in a metabolic pathway and found a strong positive correlation between most pairs of metabolites (Table S2, Supplementary information). For example, metabolites involved in the glycolytic pathway showed strong positive correlation when paired against each other. These include pairs of sucrose:fructose (r = 0.896), sucrose:glucose (r = 0.956), maltose:fructose (r = 0.95), glucose:fructose (r = 0.93), turanose:fructose (r = 0.95), turanose:glucose (r = 0.92), turanose:maltose (r = 0.96), fructose:palmitic acid (r = 0.91), and palmitic acid:glucose (r = 0.91). Similar results were observed for metabolite pairs of the TCA pathway viz. oxalic acid:malic acid (r = 0.86), malonic acid:aspartic acid (r = 0.91), malonic acid:glutamine (r = 0.91), malonic acid:benzoic acid (r = 0.96) and the shikimate pathway viz. phenylalanine:tyrosine (r = 0.93). We have also found interesting positive and negative correlations among different metabolites in a categorical manner. The major sugars which correspond to the sprouting process such as glucose, fructose, and maltose exhibited strong positive correlation with the following metabolites: palmitic acid, turanose, oxalic acid, tetronic acid, ethanolamine, linoleic acid, but was negatively correlated with mannoside, sitosterol, and octadeconoic acid. Asparagine, aspartic acid, gamma aminobutyric acid, glycine, leucine, ornithine and threonine showed strong positive correlation with hexadecanoate, altronic acid, arabinohexaric, citric acid, monopalmitin, malonic acid, benzoic acid, glutamine, glycine, lactic acid, ethanolamine, ornithine, butanoic acid, capric acid, cycloartenol, glyceric acid, heptanoic acid, margaric acid, pelargonic acid, tetronic acid, glycerol, myo-inositol and xylose. On the other hand, alanine and gamma aminobutyric acid had strong negative correlation with methylboronate and monoamidoethylmalonic acid. Myristic acid, palmitic acid, pelargonic acid, lauric acid and monopalmitin had strong positive correlation with ethanolamine, glucoside, glutaric acid, linoleic acid, fructose, galactose, glucose, heptanoic acid, maltose, tetronic acid, turanose, tryrosine, leucine, arabitol, butanoic acid, capric acid, cycloartanol, glyceric acid, glycine, margaric acid, caporic acid, docosadienoic acid, fucose, ricinoleic acid, aspartic acid, glutamine, benzoic acid and malonic acid. However, palmitic acid was negatively correlated with sitosterol, octadeconoic acid, tyrosine and xylonic acid. Lactic acid, malic acid, citric acid, succinic acid, benzoic acid showed positive correlation with gamma aminobutyric acid, asparagine, hexadecanoic acid, arabinohexaric acid, mannoic acid, aspartic acid, glutamine, malonic acid and monopalmitin. Clearly, these findings suggest that during bud growth and development, carbohydrate metabolism increased only when free amino acid accumulation increased and biosynthesis of fatty acids was greatly relied on the intermediates of carbohydrate metabolism.

## Discussion

Several promising functional genomics approaches such as transcriptomics, proteomics and metabolomics are employed to obtain a systemic and comprehensive understanding of various processes in plants^23, 26^. Currently, whole genome information of *Crocus sativus* is unavailable, which inturn restrict transcriptomic and proteomic studies. However, metabolomics do not require prior genomic information enabling the identification of sample constituents and the actual biochemical status of a plant. Despite myriad application of metabolomics in plant research, till date, there are no reports available on the metabolite profiling of Saffron corm. In the present study, we provide a comprehensive, comparative analysis of the metabolite composition of Saffron corm during five different development stages. The resulting information would help in understanding the contribution of key metabolites in the process of dormancy (endo-dormancy) and the mechanism of sprouting (para-dormancy) process of *Crocus* corm.

The present study illustrates the level of different metabolites and their relative abundance at five different developmental stages of Saffron corm and correlates them with the metabolic pathways. We found a relatively low abundance of reducing sugars viz. glucose, fructose, galactose, sorbitol, and turanose at stage 1 of *Crocus* corm development in comparison with non-reducing sugar such as sucrose. Further, at the onset of S2, up to the end of S3, there was a continuous remarkable increase in the level of these reducing sugars. Sucrose showed relatively higher abundance from S1, which then increased further at S2 gradually declined from S3 to S5. In addition, a gradual decrease in the level of reducing sugars was also observed from the onset of stage 4. The apical portion of the corm from which the sprout originates represent the part from where the samples were collected and analysed. These are the centres of meristematic activity exhibiting high rate of metabolism, thereby requiring large quantity of soluble carbohydrates to initiate sprouting. Indeed, the assimilates might be translocated to these regions from the other parts of the corm thus causing a progressive increase in the tissue^9, 31^. Therefore, sampling strategy adopted in the present study also appears to be critical in accounting for a gradual increase in soluble sugars and amino acids content of *Crocus* corm tissue. Our analysis of metabolic profile of corm at different developmental stages i.e. during dormant period till sprouting indicated that with the breakdown of starch content, total sugars increased as sprouting progressed. Similar observations were reported in potato tubers^32^ and in corms of *Crocus sativus* ^17^. Previously, it has been reported that from mid-June onwards, preceding the onset of S2, up to the onset of S5, a continuous breakdown of starch occurs and it is accompanied by a significant increase in the level of soluble sugars^17, 18^. These observations were recently corroborated by a transcriptome study of the dormant corm (and several other tissues parts), indicating that transcripts encoding key enzymes involved in starch degradation were highly expressed in the dormant corm^19^. Chrungoo *et al*.^17^ suggested that starch breakdown is directly related to the biosynthesis of non-reducing sugar sucrose along with glucose. This notion finds support from our results, where a relatively higher abundance of reducing sugars is observed at S2, increasing further at S3. With respect to sucrose, our present study also shows that the breakdown of sucrose seems to start from the onset of stage 3 as an appreciable relative abundance of glucose and fructose was noticed at stage 3 and thereafter all the sugar contents decreased at successive stages i.e. S4 and S5. Therefore, a progressive increase in the level of total sugars, specifically glucose and fructose during transition from stage 2 to stage 3 accompanied by a decline in the content of sucrose suggests that reducing monosaccharides as a result of sucrose breakdown finally reached its highest relative abundance to initiate the sprouting process and bud growth. Therefore, the present study along with the support from previous studies indicate that the synthesis of sucrose as a result of starch degradation and finally synthesis of glucose and fructose from sucrose breakdown might play a significant role in transition from dormancy to resumption of active growth in the buds (S1 to S2) to initiate the sprouting process (S2 to S3).

The significant increase in the level of organic acids like citric acid, malic acid, malonic acid and fumaric acid relative to tricarboxylic cycle and fatty acids (capric acid, heptadecanoic acid, docosadienoic acid, eliadic acid, lauric acid, linoleic acid, myristic acid, nonadeconoic acid, palmitic acid, oleic acid, pelargonic acid and stearic acid) from intermediates of glycolytic pathway was observed at S3. Our data suggest that these two biochemical pathways become active during transition from S2 to S3 as starch breakdown starts preceding the onset of stage 2 and glucose, the only product of starch degradation, enters into glycolysis and becomes metabolized through TCA cycle. The metabolic process involved in the cleavage of starch to hexose sugars is marked by an increased metabolic flux of glucose through glycolysis and TCA cycle accompanied by the production of organic acids and fatty acids possesses an important bearing on bud growth and development, particularly the sprouting process of corm.

In the case of amino acids, the present study showed a progressive increase of free amino acids from S2 with the highest enrichment at S3, but this pattern was reverted from S4 to S5. For example, GABA showed a 26.09-fold increase in accumulation at S3. Similar observations were also reported during sprouting of mung bean^22^ and germination of cereals such as wheat^33^ and barley^34^. These reports together point to the important role of GABA in the sprouting process. The study also suggest that an increase in the free amino acid pool in corm tissues due to proteolytic breakdown of some storage proteins and this free amino acid pool takes part in the biosynthesis of hydrolytic enzymes i.e., starch phosphorylase, amylase to initiate the metabolism through glycolysis and TCA cycle^35^.

Soluble protein is one of the important factors that help in growth and differentiation process of Saffron corm and our findings revealed that a remarkable enrichment of free amino acid (proline, serine, alanine, threonine, valine, phenylalanine and glycine) occurred during transition from S2 to S3. The results of the present study signified a remarkable fold change in the free amino acid pool in corm tissue that occurs due to proteolytic breakdown of some storage proteins and thus switching the glycolysis and TCA cycle. However, these metabolic events collectively influence the dormancy breakdown and sprouting process of Saffron corm.

In conclusion, metabolic profiling of a diverse set of compounds such as sugars, amino acids, fatty acids was quite evident which could detect the alteration in different metabolite contents with respect to five developmental stages of Saffron corm studied in the present work. The findings of this study are quite consistent with the changes in metabolic flux involved in dormancy break and sprouting process. Each stage of bud growth and development bears an appreciable fold change in metabolites, with the most significant fold change occurring at the transition towards the sprouting process. So, the metabolic profiling and tissue enrichment at different developmental stages, as well as their fold change during switching from one stage to another, gives a clear indication that most of the biochemical pathways essential for bud growth and development, get accelerated from S2 and showed the highest activity of transition from S2 to S3. These changes might play a significant role in sprouting process of Saffron corm during early August to early October. Taking together both RNA and protein profiling, the metabolic complement will allow a full scenario of the complexity of the biochemical entity under study.

## Methods

### Plant Material

Corms of Saffron (nearly uniform diameter of 3-4 cm) growing under field condition were harvested at monthly intervals starting early May-July (S1-dormancy), early July-August (S2-initiation and differentiation of leaf primordia), early August -late August (S3-Initiation and differentiation of floral primordia), late August-early October (S4-elongation of perianth tube) and early October-late October (S5-bud break and floral anthesis). Apical buds were excised from the corms together with a portion of surrounding tissue and were immediately frozen in liquid nitrogen and stored at −80 °C (Fig. S2, Supplementary information).

### Sample preparation

Each of the frozen corm sample (100 mg) from the different developmental stages was ground into fine powder in liquid nitrogen. Samples were then mixed with 5 ml pre-chilled solvent [acetonitrile:isopropanol:water at the ratio of 3:3:2], followed by centrifugation at 12000 g at 4 °C for 10 min. The supernatant from each sample was dried with speed vacuum. Eight biological replicates were used for each stage and ribitol (0.2 mg/ml L^−1^ in water) was used as an internal standard as done earlier^36^. Two-step chemical derivatization of the extracted metabolites was performed according to the previous protocol^24^ with few modifications. Derivatization was carried out by adding 50 µl of methoxyamine hydrochloride in pyridine and shaking at 30 °C for 90 min. Further, 100 µl of N-methyl-N-trimethyl silyl triflouro acetamide (MSTFA) was added to trimethylsilylate the polar functional groups.

### GC-MS analysis

The derivatized extracts were analyzed with a Shimadzu QP2010 series Gas Chromatography coupled with a mass selective detector at the Advanced Instrumentation Research Facility (AIRF), Jawaharlal Nehru University, New Delhi. Aliquots of each sample (1 µl) was injected into a Rtx® - 5MS (60 m × 0.25 mm × 0.25 µm) column in the split mode with a split ratio of 1:25. The temperature of the injector was maintained at 250 °C. The initial oven temperature of GC was 250 °C for 5 min and after injection it was increased to 280 °C and held for 35 min at 280 °C. Helium was used as a carrier gas with the flow rate at 1 ml min^−1^. Detection was achieved by using MS detection in electron impact mode and full scan monitoring mode (*m*/*z* 40–750). The temperature of the ion source was set at 200 °C and that of the quadrupole at 250 °C. Quadrupole mass spectrometer was used as detector. For metabolite identification, we used commercially available spectral libraries NIST08, NIST11 and Wiley08 for automated peak identification.

### Data analysis

Normalization of the obtained data was carried out to remove systematic as well as replicates variation within the samples. For this purpose, the peak areas derived from each specific metabolites (analysed compounds were normalized) by the peak area derived from an internal standard within the same chromatogram, such as ribitol^26^. This step consequently results in response ratios for all the compounds analysed. The normalized value of the identified metabolites was used for correlation and clustering (PCA and PLS-DA) analyses using MetaboAnalyst 2.0. Pearson’s correlation was set as a default setting (www.metaboanalyst.ca/). Principle component analysis (PCA) was used for an unsupervised analysis and Partial least square-discrimination analysis (PLS-DA) for a supervised analysis. A range of metabolites was selected as the variable importance in the projection (VIP) based on PLS-DA. The hierarchical clustering analysis and heat map were generated by MultiExperiment Viewer-Tm4 (MeV)^37^.

## Electronic supplementary material

Supplementary information
