# Supplementary material for: Metabolic shift in sugars and amino acids regulates sprouting in Saffron corm
Source: Sci Rep. 2017 Sep 19;7:11904. doi: 10.1038/s41598-017-10528-2 (PMC5605653; doi:10.1038/s41598-017-10528-2)
Supplement: Supplementary file 1 — Supplementary information [file 41598_2017_10528_MOESM1_ESM.doc]

**Supplementary information**

**Metabolic shift in sugars and amino acids regulates sprouting in Saffron corm**

Jayram Bagri^1^, Anupama Yadav^1^, Khalid Anwar^1^, Jeremy Dkhar^1^, Sneh Lata Singla- Pareek^2^ and Ashwani Pareek^1*^

^1^Stress Physiology and Molecular Biology Laboratory, School of Life Sciences, Jawaharlal Nehru University, New Delhi, India

^2^Plant Stress Biology, International Centre for Genetic Engineering and Biotechnology, Aruna Asaf Ali Marg, New Delhi, India

Jayram Bagri

E-mail: [jayhcu88@gmail.com](mailto:jayhcu88@gmail.com)

Anupama Yadav

E-mail: [anuyadav242@gmail.com](mailto:anuyadav242@gmail.com)

Khalid Anwar

E-mail:[khalidbiochem@gmail.com](mailto:khalidbiochem@gmail.com)

Jeremy Dkhar

E-mail:[jeremydkhar@gmail.com](mailto:jeremydkhar@gmail.com)

Sneh Lata Singla-Pareek

E-mail: [snehpareek@gmail.com](mailto:snehpareek@gmail.com)

*Corresponding author

Ashwani Pareek

Stress Physiology and Molecular Biology Laboratory,

School of Life Sciences, Jawaharlal Nehru University,

New Delhi 110067, India

Phone: +91-11-26704504

Fax No.: +91-11-26742558

E-mail: [ashwanip@mail.jnu.ac.in](mailto:ashwanip@mail.jnu.ac.in)


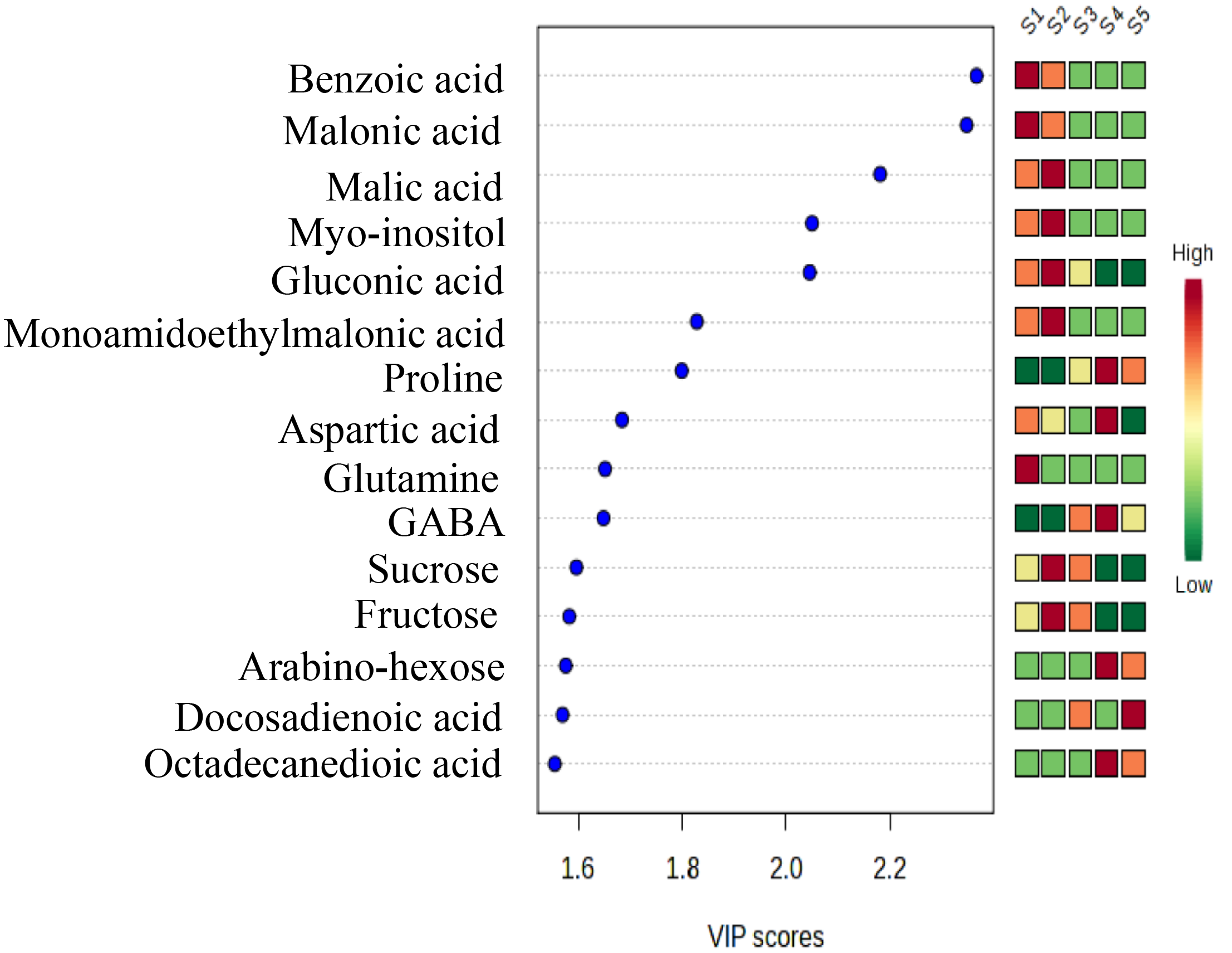


**Fig. S1.** Metabolites identified from the PLS-DA variable importance projection (VIPs) showed major differences in their accumulation in Saffron corm at five key different developmental stages (S1-S5).


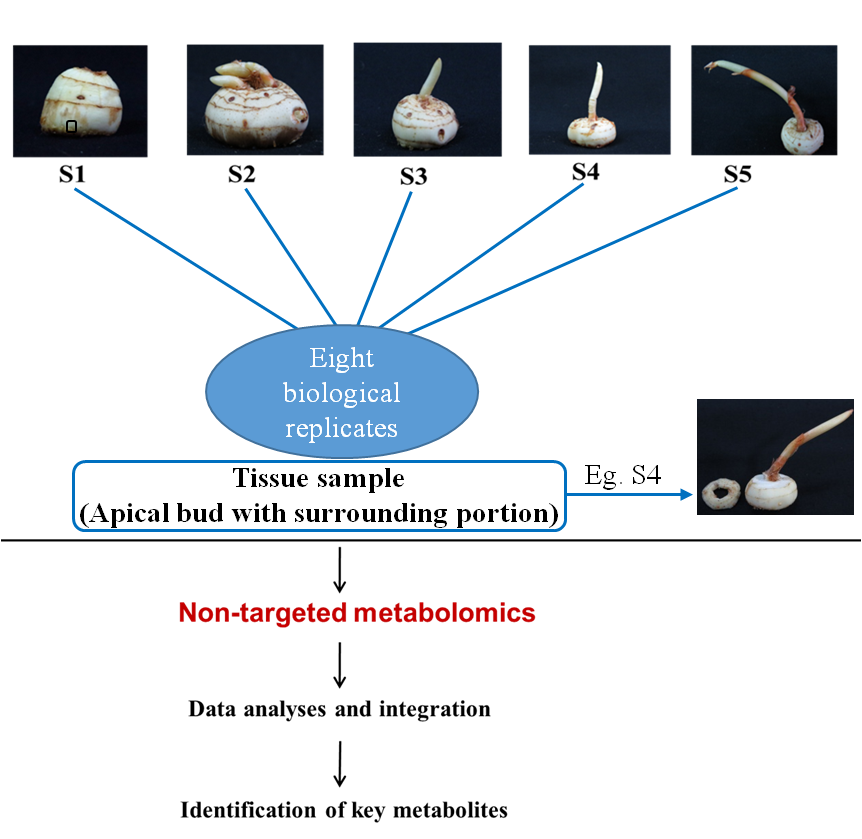


**Fig. S2.** Flow chart showing the major steps for the metabolite profiling of Saffron corm by Gas chromatography–mass spectrometry (GC-MS) carried out at five key developmental stages.

**Table S1**. Relative abundance of metabolites in *Crocus* corm tissue at five key developmental stages. Values are means (+S.E.) of eight biological replicates.

|  |  | **S1** | **S2** | **S3** | **S4** | **S5** |
| --- | --- | --- | --- | --- | --- | --- |
| **Amino acids** | **Metabolite** | **Mean±SE (n-8)** | **Mean±SE (n-8)** | **Mean±SE (n-8)** | **Mean±SE (n-8)** | **Mean±SE (n-8)** |
|  | Alanine | 0.0036061±0.00017418 | 0.0759±0.0028158 | 0.1382±0.0037005 | 0.127±0.0029716 | 0.0096895±0.00026291 |
|  | Asparagine | n.d. | 0.0252±0.0014755 | n.d. | n.d. | n.d. |
|  | Aspartic acid | 0.0359±0.00086101 | 0.052±0.0030662 | n.d. | n.d. | n.d. |
|  | GABA | 0.1161±0.0040892 | 0.1779±0.0236 | 4.6431±2.6894 | 0.715±0.3183 | 0.4013±0.2204 |
|  | Glutamine | 0.0694±0.0012776 | 0.1185±0.0066633 | n.d. | n.d. | n.d. |
|  | Glycine | 0.0097801±0.00045319 | 0.0399±0.0034888 | 0.461±0.0707 | 0.0261±0.0050842 | 0.0941±0.0543 |
|  | Leucine | n.d. | 0.0443±0.0021297 | 0.5248±0.0194 | n.d. | n.d. |
|  | Ornithine | n.d. | 0.056±0.0047598 | 0.035±0.0079282 | n.d. | n.d. |
|  | Proline | 0.6208±0.0106 | 0.4799±0.0223 | 1.3086±0.2903 | 1.1961±0.1983 | n.d. |
|  | Serine | 0.0216±0.0004731 | 0.1001±0.0047477 | 0.5405±0.0586 | 0.471±0.0352 | 0.1518±0.0445 |
|  | Threonine | n.d. | 0.0734±0.0059016 | 0.4121±0.1102 | n.d. | 0.1177±0.0674 |
|  | Valine | 0.0661±0.0010766 | 0.1153±0.0055601 | 0.8608±0.1237 | 0.7518±0.0373 | 0.2343±0.0845 |
|  | Tyrosine | n.d. | 0.033±0.0019625 | n.d. | n.d. | n.d. |
|  | Phenylalanine | n.d. | 0.0578±0.00384 | 0.0523±0.0058733 | 0.2299±0.0381 | n.d. |
|  | Lysine | n.d. | 0.0298±0.0020286 | n.d. | n.d. | n.d. |
| **Fatty acids** | Heptadecanoic acid | n.d. | n.d. | n.d. | n.d. | 0.0102±0.0024073 |
|  | Docosadienoic acid | n.d. | n.d. | 0.2117±0.0761 | 0.0562±0.0157 | 0.0207±0.0091677 |
|  | Elaidic acid | 0.2648±0.0074566 | 0.094±0.0057907 | 0.2416±0.1268 | 0.1152±0.0523 | 0.1849±0.0915 |
|  | Heptanoic acid | n.d. | n.d. | 0.0876±0.0224 | n.d. | n.d. |
|  | Lauric acid | n.d. | n.d. | 0.1557±0.0438 | n.d. | n.d. |
|  | Linoleic acid | 0.0085447±0.00040728 | 0.006863±0.00044968 | 0.115±0.0139 | 0.0697±0.0234 | 0.0276±0.0106 |
|  | Margaric acid | n.d. | n.d. | 0.0505±0.0060615 | n.d. | 0 |
|  | Monopalmitin | 0.0139±0.001098 | 0.0198±0.0083155 | n.d. | n.d. | n.d. |
|  | Myristic acid | n.d. | n.d. | 0.4178±0.1358 | 0.1668±0.0655 | 0.0553±0.0287 |
|  | Nonadecanoic acid | n.d. | n.d. | 0.006228±0.0013796 | n.d. | n.d. |
|  | Octadecanedioic acid | n.d. | n.d. | 0 | n.d. | 0.0088149±0.0019863 |
|  | Oleic acid | n.d. | 0.1217±0.0085551 | 0.918±0.2012 | n.d. | n.d. |
|  | Palmitic acid | 0.2078±0.0068436 | 0.1926±0.0090567 | 1.4193±0.0481 | 0.5837±0.1394 | 0.3271±0.085 |
|  | Palmitoleic acid | n.d. | n.d. | n.d. | 0.1157±0.0447 | n.d. |
|  | Pelargonic acid | n.d. | n.d. | 0.1064±0.0313 | n.d. | n.d. |
|  | Arachidic acid | n.d. | n.d. | 0.0693±0.0024518 | n.d. | n.d. |
|  | Capric Acid | n.d. | n.d. | 0.1032±0.0387 | n.d. | n.d. |
|  | Stearic acid | 0.0782±0.0172 | 0.0923±0.0048647 | 0.3993±0.0623 | 0.2079±0.056 | 0.1241±0.0387 |
|  | Ricinoleic acid | n.d. | n.d. | 0.7365±0.1642 | n.d. | n.d. |
| **Sugars and sugar alcohols** | Arabitol | n.d. | n.d. | 2.5957±0.0825 | n.d. | n.d. |
|  | Turanose | 0.0162±0.0012777 | 0.0561±0.0047764 | 0.1048±0.0216 | 0.039±0.0139 | 0.0282±0.0112 |
|  | Xylose | n.d. | 0.0501±0.0040701 | 0.1121±0.0415 | 0.1044±0.0262 | 0.2098±0.0615 |
|  | Sucrose | 1.2506±0.0738 | 2.2757±0.0916 | 0.9904±0.1034 | 0.8657±0.1674 | 0.5042±0.1292 |
|  | Sorbose | 0.0138±0.0030333 | 0.1426±0.0083624 | n.d. | n.d. | n.d. |
|  | Sorbitol | 0.1734±0.0318 | 0.2062±0.0129 | 0.063±0.0153 | 0.045±0.0171 | 0.0863±0.0124 |
|  | Ribose | n.d. | 0.0154±0.001019 | n.d. | 0.0494±0.0227 | 0.0235±0.0102 |
|  | Rhamnose | 0.0704±0.0107 | 0.3581±0.0116 | n.d. | n.d. | 0.1688±0.0453 |
|  | Mannose | 0.0393±0.0055343 | n.d. | 0.1858±0.0407 | 0.0509±0.0139 | 0.0671±0.0179 |
|  | Lactose | n.d. | n.d. | 0.1937±0.0552 | 0.3642±0.0668 | 0.2327±0.0492 |
|  | Maltose | 0.0135±0.00065331 | 0.0289±0.002871 | 0.1083±0.0214 | 0.0574±0.0183 | 0.0331±0.0133 |
|  | Mannoside | n.d. | n.d. | 0.0159±0.0046653 | 0.01±0.0025843 | n.d. |
|  | Melibiose | 0.0347±0.0098349 | 0.0571±0.0041428 | 0.0944±0.0208 | 0.0837±0.0175 | 0.0498±0.0159 |
|  | Arabino-Hexose | 0.0193±0.0042666 | 0.0669±0.0024615 | 0.1531±0.0338 | n.d. | n.d. |
|  | Erythrose | n.d. | n.d. | 0.0511±0.0045579 | n.d. | n.d. |
|  | Fructose | 0.1589±0.0032266 | 0.2561±0.0235 | 0.6662±0.147 | 0.8737±0.2737 | 0.1843±0.0947 |
|  | Fucose | n.d. | n.d. | 0.0196±0.0048148 | 0.0313±0.0192 | n.d. |
|  | Galactose | 0.6535±0.0221 | 0.68±0.1508 | 1.4723±0.2305 | 1.0295±0.202 | 0.8391±0.1772 |
|  | Galactoside | 0.0555±0.0459 | 1.7404±0.1776 | 0.0978±0.0119 | 0.2912±0.1733 | 0.1015±0.0511 |
|  | Glucose | 0.1935±0.0062853 | 0.6435±0.0216 | 0.9468±0.2147 | 1.2944±0.4167 | 0.2962±0.0631 |
|  | Glucoside | n.d. | n.d. | 0.4488±0.1116 | 0.4473±0.1732 | 0.1505±0.0895 |
| **Polyols** | Glycerol | n.d. | 0.7126±0.0279 | 1.2177±0.1963 | 0.6051±0.2465 | 0.1054±0.0065665 |
|  | Sitosterol | n.d. | n.d. | 0.0129±0.0030904 | 0.0113±0.00044975 | 0.0105±0.0004378 |
|  | Myo-inositol | n.d. | 0.404±0.0168 | 1.0578±0.1238 | 0.608±0.173 | 0.3093±0.115 |
| **Organic acids** | Glyceric acid | n.d. | n.d. | 0.5666±0.0822 | n.d. | n.d. |
|  | Glycolic acid | n.d. | n.d. | 0.0114±0.0030182 | n.d. | n.d. |
|  | Caproic acid | n.d. | n.d. | 0.0493±0.0173 | n.d. | n.d. |
|  | Ethanolamine | n.d. | 0.0235±0.0016452 | 0.3325±0.0474 | 0.2395±0.0862 | 0.0704±0.0401 |
|  | Hexadecanoate | n.d. | 0.0338±0.0024772 | n.d. | n.d. | n.d. |
|  | Propanol | n.d. | 0.0946±0.003903 | n.d. | n.d. | n.d. |
|  | Altronic acid | n.d. | 0.0434±0.0097333 | n.d. | n.d. | n.d. |
|  | 2,3-Butanediol | n.d. | n.d. | 0.0075604±0.0016674 | n.d. | n.d. |
|  | Aesculin | 0.007431±0.00030651 | 0.0328±0.0050826 | 0.0364±0.0080376 | n.d. | n.d. |
|  | Arabinohexaric | n.d. | 0.0124±0.00060277 | n.d. | n.d. | n.d. |
|  | Arabinonic acid | n.d. | 0.0277±0.0014089 | n.d. | n.d. | n.d. |
|  | Citric acid | n.d. | 0.0859±0.0194 | n.d. | n.d. | n.d. |
|  | Cycloartenol | n.d. | 0.0101±0.0025863 | 0.0636±0.006978 | 0.0455±0.0184 | n.d. |
|  | Butanoic acid | n.d. | n.d. | 0.0519±0.0027539 | n.d. | n.d. |
|  | Benzoic acid | 0.2373±0.0041061 | 0.1316±0.0032106 | n.d. | n.d. | n.d. |
|  | Gluconic acid | 0.0079356±0.0017483 | 0.0327±0.0076187 | n.d. | n.d. | n.d. |
|  | Glutaric acid | n.d. | n.d. | 0.1412±0.0083231 | 0.151±0.0524 | 0.0526±0.0318 |
|  | Lactic acid | n.d. | 0.042±0.002344 | 0.1192±0.0138 | n.d. | 0.0623±0.0378 |
|  | Methylboronate | 0.0338±0.0012551 | n.d. | n.d. | n.d. | 0.0192±0.0045246 |
|  | Malic acid | 0.0404±0.00071264 | 0.0863±0.0041591 | 0.0698±0.0172 | n.d. | n.d. |
|  | Malonic acid | 0.3338±0.0076846 | 0.0329±0.0015545 | n.d. | n.d. | n.d. |
|  | Mannonic acid | n.d. | 0.1181±0.018 | n.d. | 0.0452±0.0117 | n.d. |
|  | Monoamidoethylmalonic acid | 0.1035±0.005779 | n.d. | n.d. | n.d. | n.d. |
|  | Oxalic acid | 0.1325±0.00275 | 0.042±0.0019438 | 0.4557±0.221 | 0.0952±0.0197 | 0.0469±0.0181 |
|  | Ribonic acid | 0.0112±0.00035576 | 0.0311±0.0030035 | 0.0703±0.0056435 | n.d. | n.d. |
|  | Succinic acid | n.d. | n.d. | 0.0186±0.0045242 | n.d. | n.d. |
|  | Tetronic acid | 0.0114±0.0002217 | 0.0416±0.0024107 | 0.267±0.0284 | n.d. | 0.0603±0.0371 |
|  | Threonic acid | n.d. | 0.0133±0.00038885 | n.d. | n.d. | n.d. |
|  | Uridine | n.d. | n.d. | 0.0584±0.0063855 | n.d. | 0.016±0.0053376 |
|  | Xylonic acid | n.d. | 0.0052572±0.0011611 | n.d. | n.d. | n.d. |
| n.d. = not detected | | | | | | |

**Table S2**. Correlation of metabolite-metabolite interaction by Pearson correlation coefficient method in *Crocus* corm at five key developmental stages.

| **Metabolite** | **Metabolites (Person Correlation Coefficient, r≥0.8)** | |  |
| --- | --- | --- | --- |
| Alanine | Methylboronate (-0.82145) | |  |
| Asparagine | Hexadecanoate (0.99828), Altronic acid (0.83288), Arabinohexaric (0.99984), Citric acid (0.81934) | |  |
| Aspartic acid | Monopalmitin (0.94744), Malonic acid (0.91586), Benzoic acid (0.98764), Glutamine (0.99946) | |  |
| GABA | Glycine (0.82993), Lactic acid (0.8047), Monoamidoethylmalonic acid (-0.80494) | |  |
| Glutamine | Aspartic acid (0.99946), Benzoic acid (0.98618), Malonic acid (0.91097), Monopalmitin (0.95262) | |  |
| Glycine | GABA (0.92893), Ethanolamine (0.89977) | |  |
| Leucine | Ornithine (0.89235), Butanoic acid (0.83754), Capric Acid (0.80314), Cycloartenol (0.94764), Glyceric acid (0.81728), Glycine (0.83625), Heptanoic acid (0.8255), Margaric acid (0.8237), Pelargonic acid (0.82102), Tetronic acid (0.80337) | |  |
| Ornithine | Glycerol (0.80011) | |  |
| Threonine | Myo-inositol (0.83283), Xylose (0.85173) | |  |
| Hexadecanoate | Asparagine (0.99828), Altronic acid (0.82072), Arabinohexaric (0.9979), Citric acid (0.82338) | |  |
| 2,3-Butanediol | Sitosterol (0.86856), Fucose (0.86829), Mannoside (0.9225), Ribonic acid (0.8389), Ricinoleic acid (0.86724) | |  |
| Altronic acid | Asparagine (0.83288), Hexadecanoate (0.82072), Arabinohexaric (0.83595), Gluconic acid (0.84354) | |  |
| Arabinohexaric | Asparagine (0.99984), Hexadecanoate (0.9979), Altronic acid (0.83595), Citric acid (0.81179) | |  |
| Arabinonic acid | Mannonic acid (0.91858), Mannose (-0.91276), Sorbose (0.89912), Threonic acid (0.89429), Tyrosine (0.92874) | |  |
| Arabitol | Butanoic acid (0.85574), Capric Acid (0.82369), Glyceric acid (0.83694), Glycine (0.85358), Heptanoic acid (0.84509), Lauric acid (0.90243), Margaric acid (0.8433), Pelargonic acid (0.84049) | |  |
| Benzoic acid | Aspartic acid (0.98764), Glutamine (0.98618), Malonic acid (0.96566), Monopalmitin (0.95478) | |  |
| Butanoic acid | Leucine (0.83754), Arabitol (0.85574), Capric Acid (0.96858), Cycloartenol (0.86221), Glutaric acid (0.81016), Glyceric acid (0.97851), Glycine (0.99793), Glycolic acid (0.80643), Heptanoic acid (0.99078), Margaric acid (0.98495), Nonadecanoic acid (0.81689), Pelargonic acid (0.98582) | |  |
| Sitosterol | 2,3-Butanediol (0.86856), Fructose (-0.83783), Fucose (0.85233), Galactose (-0.8289), Glucose (-0871), Mannoside (0.90549), Octadecanedioic acid (0.9029), Palmitic acid (-0.881), Tryosine (0.84728), Xylonic acid (0.81233) | |  |
| Capric Acid | Leucine (0.80314), Arabitol (0.80314), Butanoic acid (0.96858), Cycloartenol (0.83412), Glyceric acid (0.9806), Glycine (0.96547), Heptanoic acid (0.9909), Margaric acid (0.9793), Pelargonic acid (0.98932) | |  |
| Caproic acid | Docosadienoic acid (0.89259), Fucose (0.90798), Lauric acid (0.95322), Mannoside (0.88363), Ribonic acid (0.8256), Ricinoleic acid (0.80692) | |  |
| Citric acid | Asparagine (0.81834), Hexadecanoate (0.82338), Arabinohexaric (0.81179) | |  |
| Cycloartenol | Leucine (0.94764), Butanoic acid (0.86221), Capric acid (0.83412), Glyceric acid (0.84017), Glycine (0.8579), Heptanoic acid (0.85756), Margaric acid (0.8508), Pelargonic acid (0.8559) | |  |
| Ethanolamine | Glycine (0.89977), Glucoside (0.821266), Glutaric acid (0.82567), Glycerol (0.80791), Linoleic acid (0.81554), Maltose (0.83354), Myo-inistol (0.9221), Myristic (0.8443), Tetronic acid (0.8467), Turanose (0.80912) | |  |
| Fructose | Sitosterol (-0.83783), Galactose (0.81292), Glucose (0.9383), Maltose (0.9524), Octadecanedioic acid (-0.849), Oxalic acid (0.9125), Tetronic acid (0.88169), Turanose (0.9559), Sucrose (0.896) | |  |
| Fucose | 2,3-Butanediol (0.8682), Sitosterol (0.85233), Caproic acid (0.90798), Docosadienoic acid (0.83356), Lauric acid (0.8111), Mannoside (0.9108), Ribonic acid (0.8387) | |  |
| Galactose | Sitosterol (-0.8028), Fructose (0.8129), Palmitic acid (0.87203), Tyrosine (-0.81232), Xylonic acid (-0.842) | |  |
| Gluconic acid | Altronic acid (0.84354) | |  |
| Glucose | Sitosterol (-0.87068), Fructose (0.93832), Galactose (0.807), Maltose (0.89381), Mannose (-0.82), Palmitic acid (0.90365), Turanose (0.9279), Sucrose (0.956) | |  |
| Glucoside | Ethanolamine (0.82126), Glutaric acid (0.95126), Linoleic acid (0.85698), Myristic acid (0.9752) | |  |
| Glutaric acid | Butanoic acid (0.81916), Ethanolamine (0.82567), Glucoside (0.95125), Glycine (0.80735), Linoleic acid (0.8836), Margaric (0.80167), Myristic acid (0.9381) | |  |
| Glyceric acid | Leucine (0.81728), Arabitol (0.83694), Butanoic acid (0.97851), Capric acid (0.9806), Cycloartenol (0.8402), Glycine (0.97509), Glycolic acid (0.8051), Heptanoic acid (0.9799), Margaric acid (0.9982), Pelargonic acid (0.97245) | |  |
| Glycerol | Ornithine (0.80011), Myo-inositol (0.94147), Aminobutyrate (-0.82732) | |  |
| Glycine | Leucine (0.83625), Arbitol (0.8538), Butanoic acid (0.99793), Capric acid (0.96547), Cycloartenol (0.8579), Glutaric acid (0.80735), Glyceric acid (0.9751), Glycolic acid (0.8061), Heptanoic acid (0.9877), Glucoside (0.981), Nonadecanoic acid (0.8139) | |  |
| Glycolic acid | Butanoic acid (0.80643), Glyceric acid (0.80512), Glycine (0.80614), Margaric acid (0.81581), Nonadecanoic acid (0.9829) | |  |
| Heptanoic acid | Leucine (0.8255), Arabitol (0.84509), Butanoic acid (0.9907), Glycine (0.99094), Cycloartenol (0.99094), Glyceric acid (0.97987), Glycine (0.9878), Margaric acid (0.9846), Pelargonic acid (0.99869) | |  |
| Lactic acid | GABA (0.8047) | |  |
| Lauric acid | Caproic acid (0.95322), Docosadienoic acid (0.86317), Fucose (0.81108), Ricinoleic acid (0.80113) | |  |
| Methylboronate | Alanine (-0.82145), Phenylalanine (-0.81109) | |  |
| Linoleic acid | Ethanolamine (0.81537), Glucoside (0.85698), Glutaric acid (0.88359), Maltose (0.82737), Mannose (0.8014), Myristic acid (0.86218), Palmitic acid (0.8768), Tetronic acid (0.8434) | |  |
| Malic acid | Arabino-Hexose (0.91098) | |  |
| Malonic acid | Aspartic acid (0.91586), Glutamine (0.91097), Benzoic acid (0.96556), Monoamidoethylmalonic acid (0.84075), Monopalmitin (0.8949) | |  |
| Maltose | Ethanolamine (0.83543), Fructose (0.9524), Glucose (0.8938), Linoleic acid (0.8273), Palmitic acid (0.8979), Tetronic acid (0.87936), Turanose (0.9607) | |  |
| Mannonic acid | Arabinonic acid (0.91858), Mannose (-0.90653), Sorbose (0.87382) | |  |
| Mannose | Arabinonic acid (-0.91276), Linoleic acid (0.80143), Mannoic acid (-0.9065), Sorbose (-0.88929), Tyrosine (-0.938) | |  |
| Mannoside | 2,3-Butanediol (0.9225), Sitosterol (0.90549), Caproic acid (0.88369), Docosadienoic acid (0.81235), Galactose (0.9109), Glucose (-0.81997), Ribonic acid (0.8604), Ricinolic acid (0.8193) | |  |
| Margaric acid | Leucine (0.8237), Arabitol (0.8433), Butanoic acid (0.9849), Capric acid (0.9793), Cycloartenol (0.8508), Glutaric acid (0.80167), Glyceric acid (0.9983), Glycine (0.981), Glycolic acid (0.81581), Heptanoic acid (0.98464), Pelargonic acid (0.9784) | |  |
| Monoamidoethylmalonic acid | | GABA (-0.80494), Malonic acid (0.84075) | |
| Monopalmitin | Aspartic acid (0.94744), Glutamine (0.95262), Benzoic acid (0.95478), Malonic acid (0.89492) | |  |
| Myo-inositol | Threonine (0.83283), Ethanolamine (0.9228), Glycerol (0.94147), Aminobutyrate (-0.85863) | |  |
| Myristic acid | Ethanolamine (0.8342), Glucoside (0.9752), Glutaric acid (0.93808), Linoleic acid (0.86218) | |  |
| Nonadecanoic acid | Butanoic acid (0.81689), Glycine (0.81391), Glycolic acid (0.98286) | |  |
| Octadecanedioic acid | Sitosterol (0.90289), Fructose (-0.84903), Palmitic acid (-0.80419) | |  |
| Palmitic acid | Sitosterol (-0.881), Fructose (0.9125), Galactose (0.8720), Glucose (0.90365), Heptanoic acid (0.8768), Maltose (0.8979), Octadecanedioic acid (-0.804), Tetronic acid (0.8195), Turanose (0.8819), Tyrosine (-0.84795), Xylonic acid (-0.87044) | |  |
| Pelargonic acid | Leucine (0.82102), Arabitol (0.84049), Butanoic acid (0.98582), Capric acid (0.98932), Cycloartenol (0.8559), Glyceric acid (0.97245), Glycine (0.9837), Heptanonic acid (0.9987), Margaric acid (0.97841) | |  |
| Phenylalanine | Methylboronate (-0.81109), Ribonic acid (0.91541), Tyrosine (0.81009) | |  |
| Ribonic acid | 2,3-Butanediol (0.8389), Caproic acid (0.82559), Fucose (0.8387), Phenylalanine (0.91541) | |  |
| Ricinoleic acid | 2,3-Butanediol (0.86724), Caproic acid (0.80692), Docosadienoic acid (0.82169), Lauric acid (0.80113), Mannose (0.8193), Succinic acid (0.84961) | |  |
| Sorbose | Arabinonic acid (0.89912), Mannonic acid (0.8738), Mannose (-0.88929), Tyrosine (0.8387) | |  |
| Succinic acid | Mannonic acid (0.84961) | |  |
| Tetronic acid | Leucine (0.80337), Ethanolamine (0.84673), Fructose (0.8168), Linoleic acid (0.84336), Maltose (0.8794), Palmitic acid (0.81494), Turanose (0.8709), | |  |
| Threonic acid | Arabinonic acid (0.89429), Tyrosine (0.8697) | |  |
| Turanose | Ethanolamine (0.80912), Fructose (0.95687), Glucose (0.9279), Maltose (0.96065), Palmitic acid (0.8819), Tetronic acid (0.87089) | |  |
| Tyrosine | Arabinonic acid (0.92874), Sitosterol (0.84738), Galactose (-0.81232), Mannose (-0.83807), Palmitic acid (-0.848), Phenylalanine (0.81009), Sorbose (0.8387), Threonic acid (0.8697), Xylonic acid (0.85191) | |  |
| Xylonic acid | Sitosterol (0.81233), Galactose (-0.84236), Palmitic acid (-0.87044) | |  |
| Xylose | Threonine (0.85173) | |  |
